# Decreased numbers of metachromatic cells in nasal swabs in Japanese cedar pollinosis following sublingual immunotherapy

**DOI:** 10.1002/iid3.314

**Published:** 2020-05-28

**Authors:** Kuninori Otsuka, Hirokuni Otsuka, Shoji Matsune, Kimihiro Okubo

**Affiliations:** ^1^ Otsuka ENT Clinic Yokohama Kanagawa Japan; ^2^ Otorhinolaryngology Shin‐yurigaoka General Hospital Kawasaki Kanagawa Japan; ^3^ Otorhinolaryngology, Nippon Medical School Musashikosugi Hospital Kawasaki Kanagawa Japan; ^4^ Otorhinolaryngology and Head and Neck Surgery Nippon Medical School Tokyo Japan

**Keywords:** eosinophil, Japanese cedar pollinosis, mast cell, subcutaneous immunotherapy, sublingual immunotherapy

## Abstract

**Background and Objective:**

Nasal symptoms were reduced following allergen‐specific sublingual immunotherapy (SLIT) for allergic rhinitis. The mechanisms underlying the effectiveness of SLIT for Japanese cedar pollinosis are poorly understood. We studied changes in the numbers of metachromatic cells, eosinophils, and neutrophils following SLIT for Japanese cedar pollinosis.

**Methods:**

Nasal swabs were taken in the preseason (*n* = 32) and in pollinosis season (*n* = 49) from subjects given sublingual drop immunotherapy for an average duration of 1.5 years. The numbers of metachromatic cells (mast cells and basophils), eosinophils and neutrophils were determined and compared with those from untreated subjects in preseason (*n* = 65) and in season (*n* = 54).

**Results:**

SLIT subjects had a significantly reduced frequency of moderate to most severe symptoms in comparison to untreated subjects in preseason (*P* < .001, the Mann‐Whitney *U* test), and (*P* < .00001) in season. Metachromatic cell counts in nasal swabs of SLIT subjects in preseason and in season were lower than those of untreated subjects (*P* = .014, the Mann‐Whitney *U* test) and (*P* = .00001) respectively. Eosinophil numbers in SLIT subjects were not significantly different than in untreated subjects in both preseason (*P* = .29) and in season (*P* = .09). However, when SLIT subjects in season were divided into those with greater than or equal to 1.5 years, or <1.5 years of SLIT duration, the degree of eosinophilia in those with SLIT greater than or equal to 1.5 years was significantly lower (*P* = .011) than in untreated patients, but not in those with SLIT less than 1.5 years (*P* = .9). There were no significant differences in neutrophil numbers in nasal swabs between untreated and SLIT subjects in preseason and in season.

**Conclusion:**

One of mechanisms underlying the effectiveness of sublingual drop immunotherapy for Japanese cedar pollinosis is a reduction of the number of metachromatic cells in preseason and in season. Eosinophilia was also reduced in season in those given SLIT for greater than or equal to 1.5 years.

## Graphical abstract

The mechanisms underlying the effectiveness of sublingual immunotherapy (SLIT) for Japanese cedar pollinosis are poorly understood. One of mechanisms is a reduction of the number of metachromatic cells and eosinophils.

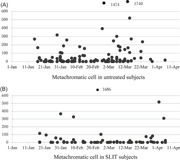

## 1 INTRODUCTION

Allergen‐specific immunotherapy for seasonal allergic rhinitis is a valuable treatment, as it can reduce the need for medications to relieve symptoms and has a long lasting effect.^1^

There are two methods of immunotherapy, subcutaneous and sublingual immunotherapy (SCIT and SLIT). Japanese cedar pollinosis is the most common form of seasonal allergic rhinitis in Japan. Since 2000, a standardized Japanese cedar pollen (JCP) liquid extract has been used for immunotherapy, and proven to be effective, by Okubo et al^2^ and Okamoto et al^3^ using SLIT drop immunotherapy in double blind, placebo‐controlled studies. In other double blind, placebo controlled studies, SLIT has been shown to be effective in seasonal allergic rhinitis to grass, ragweed, birch, and tree pollen by using standardized liquid or tablet.^4^, ^5^, ^6^, ^7^, ^8^, ^9^ Recently, a SLIT tablet for JCP has been used to establish efficacy in a double blind, placebo controlled study and is increasingly widely used.^10^, ^11^

Various mechanisms have been proposed to explain the effectiveness of SLIT. In local targeted mucosae, transforming growth factor β (TGF‐β) and Foxp3(+) were induced by interleukin 10 (IL10), and immunoglobulin G1 (IgG1) and IgG4 antibodies to pollen antigen produced by SLIT.^12^, ^13^ In peripheral blood samples, SLIT increased the numbers of regulatory T (Treg) cells, reduced of the numbers of IL4+ cells^14^ and reduced the activation of basophils.^15^ SLIT decreased nasal tryptase and eosinophil cationic protein (ECP) in season, or following allergen challenge,^16^ and there was a reduction of eosinophil numbers.^17^ However, there have been no reports of the effects of SLIT on mast cells.

We reported a reduction of the numbers of nasal metachromatic cells during HD/mite immunotherapy.^18^ Moreover, we recently reported that numbers of metachromatic cells and eosinophils were reduced in the nasal swabs of Japanese cedar pollinosis subjects in preseason and in season following SCIT compared with those of untreated subjects.^19^

In preseason and in season of 2019 we assessed symptoms and numbers of metachromatic cells, eosinophils, and neutrophils in pollinosis subjects randomized to receive JCP SLIT or not.

## 2 POLLEN COUNT

JCP counts were done every year in Yokohama by Durham method.^20^ The beginning of pollen dispersal was defined as: at least two consecutive days of 10 pollens/cm^2^ or over 20 pollens/cm^2^ on a single day. Pollen dispersal began on February 19th 2019 and counts were under 10 pollens/cm^2^ on April 16th. The cumulative JCP counts/cm^2^ in Yokohama was 3696 in 2019 (shown in Figure 2A).^19^

## 3 SUBJECTS

This study was done from 15th January until 6th April 2019. All subjects were sensitized with JCP, suffered from symptoms for over 3 to 30 years, and were diagnosed as JCP seasonal allergic rhinitis according to Japanese Guidelines.^21^, ^22^ Before the study, during pollen season they had typical symptoms and eosinophilia in nasal swabs. They were positive for IgE antibodies to JCP with class 1 or more reactivity. Subjects positive for IgE antibodies to house dust mite under class 1 and without perennial symptoms, and subjects positive for IgE antibodies to orchard grass under class 4, but with no symptoms in orchard season were included. Subjects were negative for IgE antibodies to ragweed, mugwort and Alternaria. All subjects provided informed consent. The study was approved by the Ethics Committee of Nippon Medical School.

Thirty‐one Japanese cedar pollinosis were treated with SLIT during the preseason and/or in season without interruption, and 65 untreated subjects served as controls in preseason, and 54 different individuals as controls in season (Table 1). SLIT subjects started the treatment from 2015 to 2018 and continued during off‐season and in season without interruption. Thirty of 31 SLIT subjects had their first clinic visit in preseason, and one had their first visit in season (Table 2). Two of 31 SCIT subjects had their second clinic visit in preseason and 29 in season. Eighteen had a third visit in season. In total there were 32 visits in preseason and 49 visits in season for those who received SLIT. Of the untreated subjects, 65 visited the clinic in preseason and 54 visited in season for prophylactic and symptomatic treatment using pharmacotherapies. Until the SLIT control subjects visited our clinic they did not receive any medication (confirmed through questionnaire). All SLIT subjects agreed not to take any medication, even if they had symptoms, until the study was completed. They received medications following study completion if needed.

**Table 1:** Characteristics of subjects in the sublingual immunotherapy and untreated groups

	SLIT		Untreated		
	Preseason + season	Preseason	*P* value	In season	*P* value
Number	31	65		54	
Age ± SE	45.1 ± 2.4 (15‐70)	45.8 ± 1.7 (16‐68)	0.9	41.1 ± 2.2 (16‐78)	0.15
M/F	19:12	22:43	0.0001	27:27	0.01
Class of IgE antibody	1 2 3 4 5 6	1 2 3 4 5 6		1 2 3 4 5 6	
AntiJCP IgE	0 5 18 7 1 0	0 12 25 18 8 3	0.8	1 10 27 9 6 1	0.16
Anti‐Orchard grass IgE	2 2 3 0 0 0	0 2 1 1 0 0	0.02	3 4 0 1 0 0	0.3

*Note*: There were significant differences in ages among the groups of SLIT and untreated subjects in preseason and season (*P* = .0001, *P* = .01). There were no significant differences in age and classes of anti‐JCP IgE among SLIT and untreated subjects in preseason and in‐season but a significant difference in classes of anti‐Orchard grass IgE among SLIT and untreated subjects in preseason.

Abbreviation: SLIT, sublingual immunotherapy.

**Table 2:** Frequency of clinic visits in sublingual immunotherapy and untreated groups

	SLIT		Untreated	
	31		119	
Subject numbers	January 15‐February 18 in preseason	February 19‐April 6 in season	January 15‐February 18 in preseason	February 19‐April 6 in season
Patients number (total no.)	32	49	65	54
First visit	30	1	65	54
Second visit	2	29		
Third visit		18		
Fourth visit		1		

Abbreviation: SLIT, sublingual immunotherapy.

## 4 METHODS

### 4.1 Sublingual immunotherapy

In the SLIT group, subjects commenced 0.2 mL of 200 JAU/mL (Shidatoren® Torii Co, Japan) dropped under the tongue for 2 minutes for the first 2 days, then 0.4 mL for day 3 and 4, 0.6 mL for day 5, 0.6 mL for day 6, and 1 mL for the 7th day. They then dropped 0.2 mL of 2000 JAU/mL under the tongue for day 8 and 9 day, 0.4 mL for day 10 and 11, 0.6 mL for day 12, 0.8 mL for day 13 and 1 mL for day 14. Then they continued to drop 1 mL every day. They were on SLIT everyday all year long (Table 3).

**Table 3:** Duration of Sublingual immunotherapy in 31 subjects

Average of duration ± SE, y	1.5 ± 0.2				
Duration, y	0.23‐0.49	0.5‐0.9	1‐1.49	1.5‐1.9	2.0‐3.9
Patient number	7	5	4	9	6

### 4.2 Subjects’ symptoms and severity grading

All subjects completed a detailed symptoms’ questionnaire each visit. Classification of symptom severity was done using the Japanese Guidelines.^21^, ^22^ Frequency of sneezing attacks and of blowing the nose/day were classified: none, −; 1 to 5, +; 6 to 10, ++; 11 to 20, +++; and over 21, ++++. The severity of symptoms was a cumulative score for 1 week before the clinic visit.

Nasal obstruction was classified as: none, −; feeling of obstruction but not requiring mouth breathing, +; strong obstruction requiring mouth breathing sometimes a day, ++; serious obstruction requiring mouth breathing most of a day, +++; and serious obstruction for the entire day, ++++. Severity grading was classified as: most severe; sneezing attacks or blowing the nose/day ++++ or nasal obstruction ++++, severe; sneezing attacks and blowing the nose/day +++ or nasal obstruction +++, moderate; sneezing attacks or blowing the nose/day ++ or nasal obstruction ++, mild; sneezing attacks or blowing the nose/day + or nasal obstruction +, none; no symptoms.

### 4.3 Analysis of cell types in nasal swabs

A single nasal swab was taken by cotton applicator (tip 2 × 10 mm) and transferred to a glass slide for each subject. The slides were dried and the cells were fixed in 99% methanol for 3 minutes. They were stained using Hansel solution (Torii Co, Tokyo) for 1 minute.^23^ Neutrophils and eosinophils were mainly observed in the mucous compartment of the swabs, whereas metachromatic cells were observed among epithelial cells. The total numbers of neutrophils, eosinophils and metachromatic cells (Mc) on each glass slide were assessed by a single trained observer (HO and KO) in a blinded fashion using a microscope and ×200 magnification.^24^, ^25^, ^26^ The numbers of neutrophils and eosinophils were graded as: −, none; ±, few scattered cells; 1+, found cells easily; 2+, abundant cells, with small clumps; 3+, abundant cells, often in clumps. The numbers of Mc the entire slide were counted and classified as: 0, 1 to 9 cells, 10 to 99 cells, 100 to 999 cells, greater than or equal to 1000 cells (Figure 2A,B).

### 4.4 Statistical analyses

For comparison of groups, a *t* test for age, a *χ* ^2^ test for sex ratio and the Mann Whitney *U* test for IgE class, symptom severity and cell numbers were used. *P* < .05 was considered statistically significant.

## 5 RESULTS

### 5.1 Background of subjects

There was no significant difference in ages among the groups of SLIT and untreated subjects in preseason and in season. However, the sex ratio among SLIT patients (*n* = 31) was significantly different from among untreated patients (*n* = 65) in preseason (*P* = .0001) and among intreated patients (*n* = 54) in season (*P* = .01). However, we have no evidence for differences in effectiveness of SLIT or on metachromatic cell and eosinophil numbers between sexes (data not shown). There were no significant differences in classes of IgE anti‐JCP among SLIT and untreated subjects in preseason and in season (Table 1).

### 5.2 Comparison of severity of symptoms between SLIT and untreated subjects in preseason and in season

**Figure 1:**
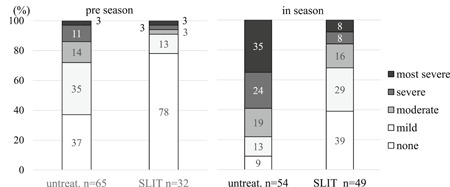
Symptom severity in untreated and SLIT subjects in preseason and season. There were significant differences in symptom severities between untreated and SLIT subjects (*P* = 0.001, the Mann‐Whitney U test) in preseason, and (*P* = 0.00001) in season

In preseason 91% of SLIT subjects had none or mild symptoms, whereas 72% of untreated subjects had none to mild symptoms (*P* = .001). In season 68% of SLIT subjects had none or mild symptoms and 32% of them had moderate to most severe symptoms. By contrast, in subjects not given SLIT, 22% had none to mild symptoms and 78% of them had moderate to most severe symptoms (*P* = .00001) (Figure 1).

### 5.3 Nasal cytology between SLIT and untreated subjects

**Figure 2:**
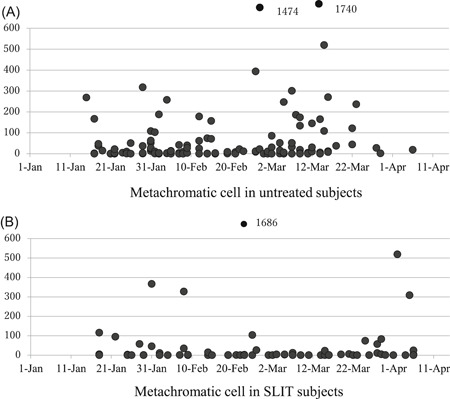
(A) Total metachromatic cell count in entire grass slide of untreated patients and (B) SLIT subjects. Pollen dispersal began on February 19th 2019

**Figure 3:**
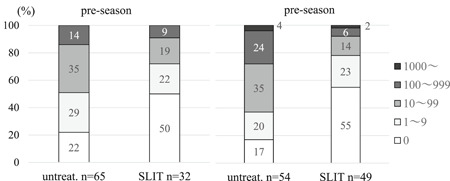
Proportion of untreated and SLIT subjects with total mast cell counts in the nasal swabs in preseason and season. There were significantly differences in metachromatic cells counts between untreated and SLIT subjects (*P* = .014, the Mann‐Whitney *U* test) in preseason, and (*P* = .00001) in season. SLIT, sublingual immunotherapy

Total Mc counts in untreated (Figure 2A) and SLIT subjects (Figure 2B) in preseason and in season show the marked reduction of nasal Mc in the SLIT subjects. In preseason, proportions of untreated subjects with total Mc number in the following categories 0, 1 to 9, 10 to 99, 100 to 999, greater than or equal to 1000 cells were 22%, 29%, 35%, 14%, and 0%, whereas these proportions in SLIT subjects were 50%, 22%, 19%, 9%, and 0% (Figure 3). There were significantly differences in number of Mc between untreated and SLIT subjects (*P* = .014) in preseason.

On the other hand, in season the proportions of untreated subjects with total Mc numbers in categories 0, 1 to 9, 10 to 99, 100 to 999, greater than or equal to 1000 cells were 17%, 20%, 35%, 24%, and 4%. In SLIT subjects the proportions were 55%, 23%, 14%, 6%, and 2%. There were significant differences in number of Mc between untreated and SLIT subjects (*P* = .00001) in season.

**Figure 4:**
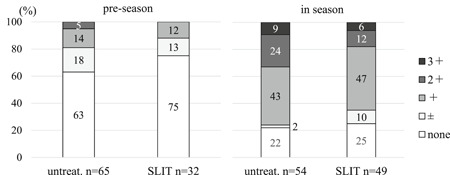
Proportion of untreated and SLIT subjects with various levels of eosinophilia in preseason and season. There was no significant difference in degrees of eosinophilia between untreated and SLIT subjects in preseason (*P* = .29, the Mann‐Whitney *U* test). The degree of eosinophilia in SLIT patients was not significantly different than in untreated subjects (*P* = .09) in season. SLIT, sublingual immunotherapy

**Figure 5:**
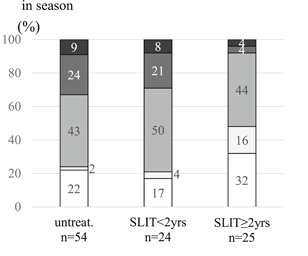
When SLIT subjects in season were divided into greater than or equal to 1.5 years (*n* = 25) and less than 1.5 years (*n* = 24) for duration of SLIT, degree of eosinophilia in subjects with SLIT greater than or equal to 1.5 years was lower than SLIT less than 1.5 years (*P* = .029) and untreated subjects (*P* = .011), whereas there was no significant difference between SLIT less than 1.5 years and untreated subjects (*P* = .9). SLIT, sublingual immunotherapy

Proportion of subjects with eosinophils none to 3+ in nasal swabs (Figures 4 and 5).

There was no significant difference in degrees of eosinophilia between untreated and SLIT subjects in preseason (*P* = .29, the Mann‐Whitney *U* test). The degree of eosinophilia in the total of the SLIT subjects was not statistically different (*P* = .09) than in untreated subjects in season (Figure 4).

However, when SLIT subjects in season were separated by duration of SLIT treatment into greater than or equal to 1.5 years (*n* = 25) and less than 1.5 years (*n* = 24), the degree of eosinophilia in subjects with SLIT greater than or equal to 1.5 years was lower than SLIT less than 1.5 years (*P* = .029) and in group of untreated subjects (*P* = .011) (Figure 5), whereas there was no significant difference between SLIT less than 1.5 years and untreated subjects (*P* = .9).

**Figure 6:**
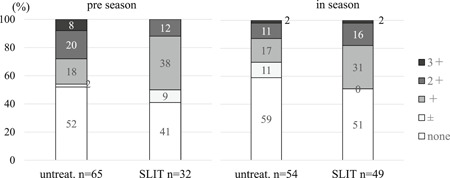
Proportion of untreated and SLIT subjects with degree of neutrophilia none to 3+ in preseason and season. There were no significant differences in degrees of neutrophilia between untreated and SLIT subjects in preseason (*P* = .88) and in season (*P* = .22). SLIT, sublingual immunotherapy

Proportion of subjects with neutrophils; none to 3+ in nasal swabs (Figure 6).

There were no significant differences in degrees of neutrophilia between untreated and SLIT subjects in preseason (*P* = .88) and in season (*P* = .22).

## 6 DISCUSSION

In 2008 over 30% of Japanese suffered from JCP pollinosis.^21^, ^22^ The number of patients is considered to be increasing. In 2018, the Welfare Department of Health published that 48.8% of citizens in Tokyo had JCP pollinosis.^27^ JCP pollinosis patients suffer symptoms from mid February to the end of April every year.

As we outlined recently,^19^ before 2000, subcutaneous immunotherapy used a conventional extract with JCP allergen containing mainly protein Cry J2 (CryJ1, 0.179 μg/mL and CryJ2, 2.39 μg/mL). This extract did not have satisfactory therapeutic efficacy.^28^, ^29^ An extract of Cry J1 was reported to be more related to skin sensitivity than that of Cry J2.^30^ In 2000, the conventional extract was replaced by standardized extract of JCP containing 7.3 to 21 μg/mL of Cry J1 (10 000 JAU/mL; Torii Co, Japan).^31^ This standardized extract has been used for SCIT in Japan and has been shown to have more substantial therapeutic effect than the older, conventional extract.^32^

Okubo et al^2^ and Okamoto et al^3^ used the Cry J1 SLIT drop therapy (2000 JAU/mL) in a double blind controlled studies and demonstrated its effectiveness. The symptom severity in SLIT subjects in preseason and in season was reduced compared to in untreated subjects. Recently, a higher dose SLIT tablet (5000 JAU) has become available and may be more effective than the lower dosage of 2000 JAU.^10^, ^11^

In our current study, we compared metachromatic cell, eosinophil and neutrophil numbers in nasal swabs of SLIT subjects and untreated subjects in preseason and in season. There were significant differences in total Mc number between SLIT patients and untreated patients in preseason (*P* = .014) and in season (*P* = .00001). There was no significant difference in the degree of eosinophilia between untreated and SLIT subjects in preseason (*P* = .29) and in season (*P* = .09). However, when SLIT subjects in season were divided into greater than or equal to 1.5 years (*n* = 25) and less than 1.5 years (*n* = 24) for duration SLIT, the degree of eosinophilia in subjects with SLIT greater than or equal to 1.5 years was lower than SLIT less than 1.5 years (*P* = .029). Further, the degree of eosinophilia in subjects given SLIT greater than or equal to 1.5 years was significantly lower (*P* = .011) than in untreated subjects, whereas there was no significantly difference between SLI less than 1.5 years and untreated subjects (*P* = .9). Degrees of neutrophilia were not significantly different between SLIT and untreated subjects in preseason and in season.

The number in Mc and eosinophils in nasal scrapings of JCP subjects was increased in season and throughout much of the year, but did often disappear by January of the following year.^33^

In this study, we showed the Mc number of nasal swabs decreased in SLIT. This might mean that Mc and eosinophils were reduced throughout the year. The mechanisms underlying the effectiveness of JCP SLIT may include induction of Treg cells, defined as the proportion of IL‐10(+) Foxp3(+) cells among CD25(+) CD4(+) leukocytes.^34^, ^35^ Mechanisms to explain the effectiveness of SLIT in other pollinosis include: increases in IL10, TGF‐β, and Foxp3(+) cells in the target mucosa, an increase in IgG1and IgG4,^12^, ^13^ reductions of IL4+ cells,^14^ reduced the activation of basophils.^15^ SLIT decreased nasal tryptase and ECP in season, or following allergen challenge,^16^ and there was a reduction of eosinophil numbers.^17^ Recently, it has also been shown that SLIT results in an increase in IL35 and inhibition of IL4 and IL13, as well as IgE production.^36^

However, there is no previous reports on the reduction of the number of nasal Mc or eosinophils during SLIT for JCP, although we recently reported that JCP SCIT reduced nasal Mc and eosinophil numbers.^19^ We previously reported the reduction of Mc number during HD/mite SCIT^18^ and Nouri‐Aria reported the reduction of IL9 and mast cells by grass pollen SCIT.^37^ The current report is thus the first report of the effects of JCP SLIT on nasal Mc and eosinophil numbers.

In humans, mast cell proliferation is directed by stem cell factor (SCF).^38^, ^39^ Previously, we^40^ and others^41^ reported that nasal epithelial cells express SCF messenger RNA and protein and that this correlated with mast cell numbers in the nasal scrapings. This mast cell proliferation by SCF is enhanced by other cytokines produced from Th2 cells, including IL‐4 and IL‐9.^42^, ^43^ Reductions of IL4, IL9, and IL5 might be responsible for the reduction of metachromatic cells and eosinophils in nasal swabs following SLIT. In turn, reductions of Mc and eosinophils may be directly related to symptom improvement.

## CONFLICT OF INTERESTS

The authors declare that there are no conflict of interests.

## Data Availability

The authors confirm that the date supporting the findings of this study are available within the article and on request from the corresponding author.
